# *α*‐Conotoxin M1 (CTx) blocks *αδ* binding sites of adult nicotinic receptors while ACh binding at *αε* sites elicits only small and short quantal synaptic currents

**DOI:** 10.14814/phy2.12188

**Published:** 2014-12-11

**Authors:** Josef Dudel

**Affiliations:** 1 Institut für Neurowissenschaften, Technische Universität München, Biedersteinerstr. 29, München, D‐80802, Germany

**Keywords:** binding site specificity, CTx block transmission, mouse nicotinic receptor, presynaptic muscarinic receptors

## Abstract

In ‘embryonic’ nicotinic receptors, low CTx concentrations are known to block only the *αδ* binding site, whereas binding of ACh at the *αγ*‐site elicits short single channel openings and short bursts. In adult muscles the *αγ*‐ is replaced by the *αε*‐site. Quantal EPSCs (qEPSCs) were elicited in adult muscles by depolarization pulses and recorded through a perfused macropatch electrode. One to 200 nmol L^−1^ CTx reduced amplitudes and decay time constants of qEPSCs, but increased their rise times. CTx block at the *αδ* binding sites was incomplete: The qEPSCs still contained long bursts from not yet blocked receptors, whereas their average decay time constants were reduced by a short burst component generated by ACh binding to the *αε*‐site. Two nanomolar CTx applied for 3 h reduced the amplitudes of qEPSCs to less than half with a constant slope. The equilibrium concentration of the block is below 1 nmol L^−1^ and lower than that of embryonic receptors. CTx‐block increased in proportion to CTx concentrations (average rate 2 **×** 10^4^ s^−1^·mol^−1^ L). Thus, the reactions of ‘embryonic’ and of adult nicotinic receptors to block by CTx are qualitatively the same. – The study of the effects of higher CTx concentrations or of longer periods of application of CTx was limited by presynaptic effects of CTx. Even low CTx concentrations severely reduced the release of quanta by activating presynaptic M2 receptors at a maximal rate of 6 **×** 10^5^ s^−1^·mol^−1^ L. When this dominant inhibition was prevented by blocking the M2 receptors with methoctramine, activation of M1 receptors was unmasked and facilitated release.

## Abstract (precis)

When CTx blocks the *αδ* binding site of adult nicotinic receptors, very small and short quantal synaptic currents (qEPSCs) are generated by binding of ACh quanta at the *αε*‐site, This is very similar to the effects of CTx at embryonic receptors where the short qEPSCs are generated by binding at the *αγ* site. CTx also activates presynaptic muscarinic M1 and M2 receptors.

## Introduction

**Figure 1.**
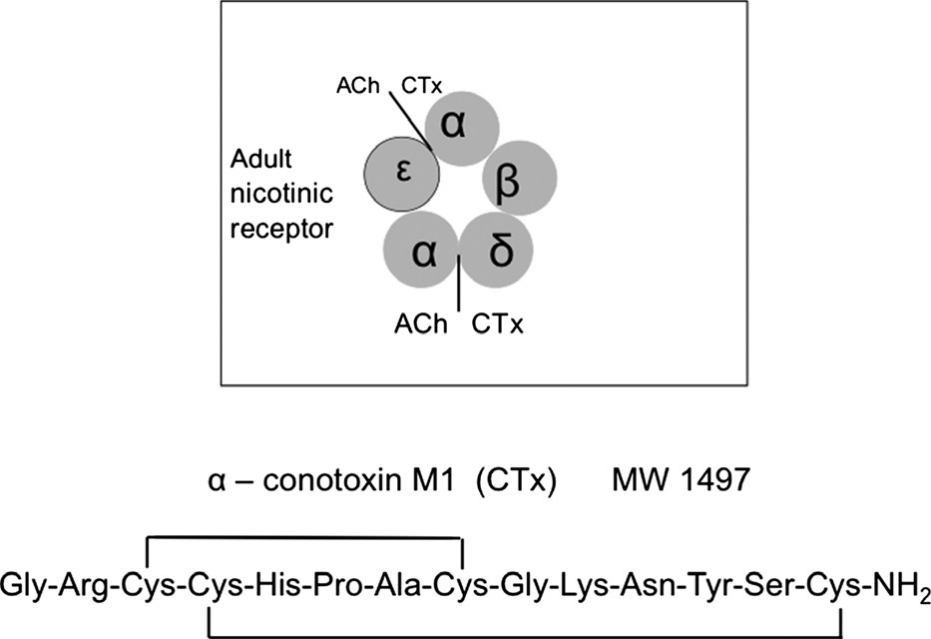
Scheme of the composition of subunits of the adult postsynaptic nicotinic ACh receptor of mouse muscle. The *αδ* binding site of the receptor has a higher affinity for ACh as well as for the blocker *α*‐conotoxin M1 (CTx) than the *αε* binding site, as indicated by the larger printing of the ligands at the *αδ* binding site. Below the composition of CTx.

Vertebrate muscles are activated by the release of quanta of acetylcholine (ACh) from the motor nerve terminal. ACh molecules bind to nicotinic receptors in the muscle membrane that intermittently open channels, and current flow depolarizes the membrane. The postsynaptic nicotinic ACh receptors are pentamers of closely related subunits that are arranged around a central pore which can open to pass channel currents (Mitra et al. 1989). The subunits are named *γαβδα* in embryonic mouse muscle and *εαβδα* in adult muscle. The two binding sites for ACh are between the *α* and the *γ* or *ε* subunits and between the *α* and the *δ* subunits (Karlin 1993; Sine et al. 1995) (Fig. 1). Binding of agonists to one of these sites has been shown to elicit several types of single short channel openings, whereas simultaneous binding to both sites elicits long bursts of channel openings. These bursts allow the flow of currents across the membrane that are sufficient to depolarize the muscle and produce neuromuscular transmission (Colquhoun and Sakmann 1985; Hallermann et al. 2005).

While the generation of long bursts of channel currents seemed to be understood, it has been possible only recently to relate several types of openings to the binding of agonists at one of the single binding sites of the embryonic nicotinic receptors. Stock et al. (2014) have compared the effects of some agonists that differ in their affinity to the *αγ*‐ and the *αδ*‐sites of patch‐clamped embryonic receptors. Binding of agonist to the *αδ* site generated single openings of on average 6 *μ*s duration. Binding to the *αγ*‐site elicited single 62 *μ*s openings, but also short, on average 0.75 ms bursts of openings. Simultaneous binding to both sites elicited the familiar long bursts of channel openings.

Stock et al. used binding site specific agonists, but also a specific blocker. The venoms of some marine cone snails, especially the fraction *α*‐conotoxin M1 (for short CTx), block neuromuscular transmission. CTx has been shown to bind primarily to the *αδ* interface of the nicotinic receptor (Fig. 1) (Sine et al. 1995; Cortez et al. 2007; Azam and McIntosh 2009). Stock et al., applying relatively high CTx concentrations to patches of membrane, found that CTx almost completely blocked ACh elicited channel openings, and only 62 *μ*s single openings and short bursts of openings remained. Such activity is elicited by binding of ACh at the *αγ*‐site, as expected when the *αδ*‐site is blocked. Stock et al. presented also synaptic quantal currents that were measured via a macropatch electrode from an ‘embryonic’ mouse muscle. CTx reduced the amplitude of qEPSCs, lengthened their rise time and shortened their decay time constant, as expected.

This study was done in parallel to the Stock et al. patch‐clamped investigation related above. While they used embryonic‐type nicotinic receptors from mouse muscle containing the *αγαδβ*‐subunits, we worked with adult mouse muscles in which the *γ*‐subunit is exchanged by an *ε*‐subunit (Fig. 1) (Dudel and Heckmann 2002). We wanted to observe the time course and concentration dependence of the effects of CTx on postsynaptic currents and to compare them to the results of the investigations at embryonic receptors. Unexpectedly, we encountered additional profound presynaptic actions of CTx on the release of transmitter quanta.

## Materials and Methods

Experiments were performed on about 20‐day‐old balb C mice that were reared in the animal department of the Institute. They were killed by cervical dislocation and decapitation. The diaphragms were excised rapidly, and hemidiaphragms were pinned down in a narrow Perspex chamber, lighted through a glass bottom and were superfused rapidly. The superfusate was saturated with 95% O_2_ and 5% CO_2_, and contained (in mmol L^−1^): NaCl, 108; KCl, 3.5; CaCl_2_, 1.5; MgCl_2_, 0.7; NaHCO_3_, 26; NaH_2_PO_4_, 1.7; Na‐gluconate, 10; glucose, 5.5; saccharose, 7.6 (pH adjusted to 7.4 adding NaOH), tetrodotoxin (TTX), 0.2 *μ*mol L^−1^. It was held at 20°C by driving it through a thermostated heat exchanger. The study employed 51 mice and recorded successfully from 35 preparations. It was approved ethically by the respective authorities.

Excitatory postsynaptic currents (EPSCs) were recorded by means of a macropatch pipette with a 10 *μ*m internal tip diameter. The tip was perfused with a solution containing (mmol L^−1^): NaCl, 162; KCl, 5.3; CaCl_2_, 2; MgCl_2_, 1; NaH_2_PO_4_, 0.67; Hepes, 15: glucose, 5.6. pH was adjusted with NaOH to 7.4 and 0.2 *μ*mol L^−1^ TTX was added to prevent excitations. The fluid at the tip was exchanged several 100 times per second.

Both the perfusate of the electrode and the superfusate of the bath could separately be switched to drug containing solutions; we used *α*‐conotoxin M1 (CTx) and the blockers of muscarinic receptors M1 and M2, pirenzepine and methoctramine, respectively.

The pipette contained a voltage‐clamp current input for synaptic currents, and a current‐clamp output for the application of depolarizing pulses. The pulses lasted 1 ms similar to the action potential and the rate of pulses was 5 s^−1^. The tip of the pipette was lowered to touch the surface of the muscle causing a slight indentation. Negative pressure of about 0.01 bar at the tip of the pipette improved the seal toward the solution outside. While applying depolarizing pulses, the tip was moved slowly over the surface of the muscle until signals from a superficial endplate were picked up. The position was optimized to obtain maximal amplitudes and minimal rise times of quantal EPSCs (qEPSCs). For further details of the recording conditions see Dudel (1983, 1989, 2007).

The recorded data were digitized at 50 kHz and stored on computer disks. The amplitudes, rise times, and decay time constants of qEPSCs were evaluated automatically by means of the modified ISO2 program (M. Friedrich, Niederhausen; see Dudel 2006). The rise times of qEPSCs were measured from 10 to 90% of the amplitude. The delays of released quanta were measured from the beginning of the depolarization pulse to the instant when the qEPSC rose to 10% of its amplitude. Average rates of release (m) were determined by counting quanta.

## Results

### Postsynaptic effects of CTx

**Figure 2.**
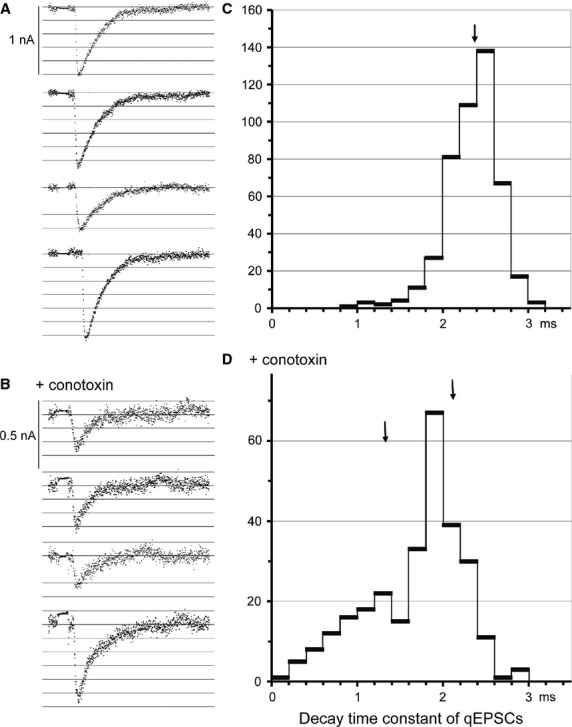
(A and B) Samples of original recordings of quantal excitatory currents (qEPSCs) by means of a macropatch electrode. The length of each trace is 20 ms. The EPSCs were elicited by 1 ms, 5 s^−1^ negative pulses through the recording electrode, see the compensated pulse artifact preceding the qEPSCs. The traces were filtered at 13 kHz. A control, B after partial block by 20 nmol L^−1^ CTx, note the doubled amplification. C and D distributions of decay time constants of the qEPSCs that were measured in the same experiment. The arrows indicate the average durations of the decay time constants.

Figure 2 shows a typical selection of single quantal postsynaptic currents (qEPSCs). At this synaptic site the control qEPSCs had average amplitudes of 1.06 ± 0.15 nA (SD, *N* = 460), a rise time of 0.36 ± 0.07 ms and a time constant of decay of 2.35 ± 0.26 ms. The qEPSCs in Fig. 2A had amplitudes from 0.65 to 1.26 nA and decay time constants from 2.4 to 2.9 ms. When 20 nmol L^−1^ CTx had been applied for 85 min, the amplitude of qEPSCs was on average maximally depressed to 0.41 ± 0.084 nA (*N* = 284), and its rise time increased to 0.46 ± 0.11 ms. The samples in Fig. 2B had amplitudes from 0.20 to 0.78 nA and decay time constants from 0.37 to 1.9 ms. The distribution of the decay time constants presented two components, a main one of 1.96 ± 0.24 ms and one about half as large (by area) of 1.03 ± 0.47 ms (Fig. 2C and D).

In the experiment of Fig. 2, block by CTx at the *αδ*‐site of the receptors obviously was quite incomplete: most qEPSCs in Fig. 2B contained long bursts of channel currents generated by doubly liganded receptors, and the larger component of the distribution of decay time constants in Fig. 2D also was derived from qEPSCs with a large contribution of long burst currents. A more clear‐cut result would be expected if the block by CTx would have been continued to become more complete. However, the presynaptic inhibition of release by CTx (Fig. 7) severely reduces the number of recorded qEPSCs necessary for a distribution of decay time constants.

The high‐resolution recordings in Fig. 2B show some curious positive deflections with varying delays after the qEPSCs; the most prominent one is seen toward the end of the lowest trace. These positive deflections are generated by EPSCs that are elicited at synaptic sites outside of the recording electrode that draw ‘electrotonic’ current from the patch‐clamped region. Due to their more distant origin they are reduced in amplitude and their time course is smoothed. Such extra‐electrode positive EPSCs are not uncommon. When selecting a recording site, they often can be abolished by slight shifts of the electrode. They are seen relatively often after application of CTx. Very rarely they are almost as large and short as the regular negative qEPSCs and then should be generated just outside of the electrode.

#### Block of qEPSCs and its recovery

**Figure 3.**
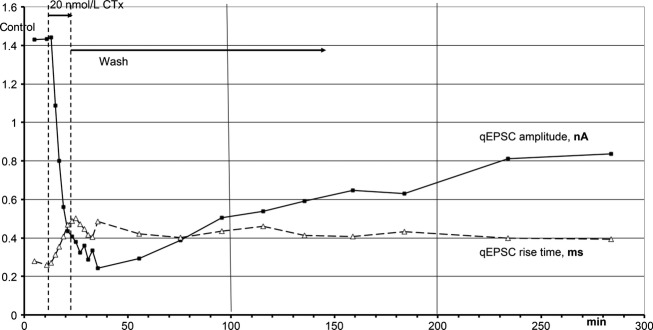
Time course of the effects of 20 nmol L^−1^ CTx on the amplitude and the rise time of qEPSCs. CTx was applied for 11 min to a synaptic site through the recording and stimulating electrode, after which the perfusate of the electrode was switched back to control solution (wash). The bath containing the muscle was superfused with control solution throughout. The ordinate scale applies for the amplitude (nA) and for the rise time (ms).

A typical time course of the effects of low CTx concentrations is shown in Fig. 3. 20 nmol L^−1^ CTx was applied for 10 min, and the amplitude of quantal postsynaptic currents (qEPSCs) was reduced from 1.4 nA to 0.24 nA. As in all such CTx applications, the effect outlasted the application considerably, here by 12 min. This delay is not entirely due to slow outwash of CTx: during the recordings presented in Fig. 3, the presynaptic depression of release by CTx started to recover within 4 min (Fig. 7).

After the peak depression of the amplitudes of the qEPSCs (at 35.5 min in Fig. 3), the amplitudes recovered to 0.84 nA within 249 min, a rate of 0.0015% of control per minute. As will be discussed below, this is not a real recovery. In parallel to the decrease of the qEPSC amplitude due to CTx, the rise time of the qEPSC increased from 0.27 to 0.5 ms and recovered even more slowly than the amplitudes. Similar increases in the rise times due to CTx were seen in all such experiments.

**Figure 4.**
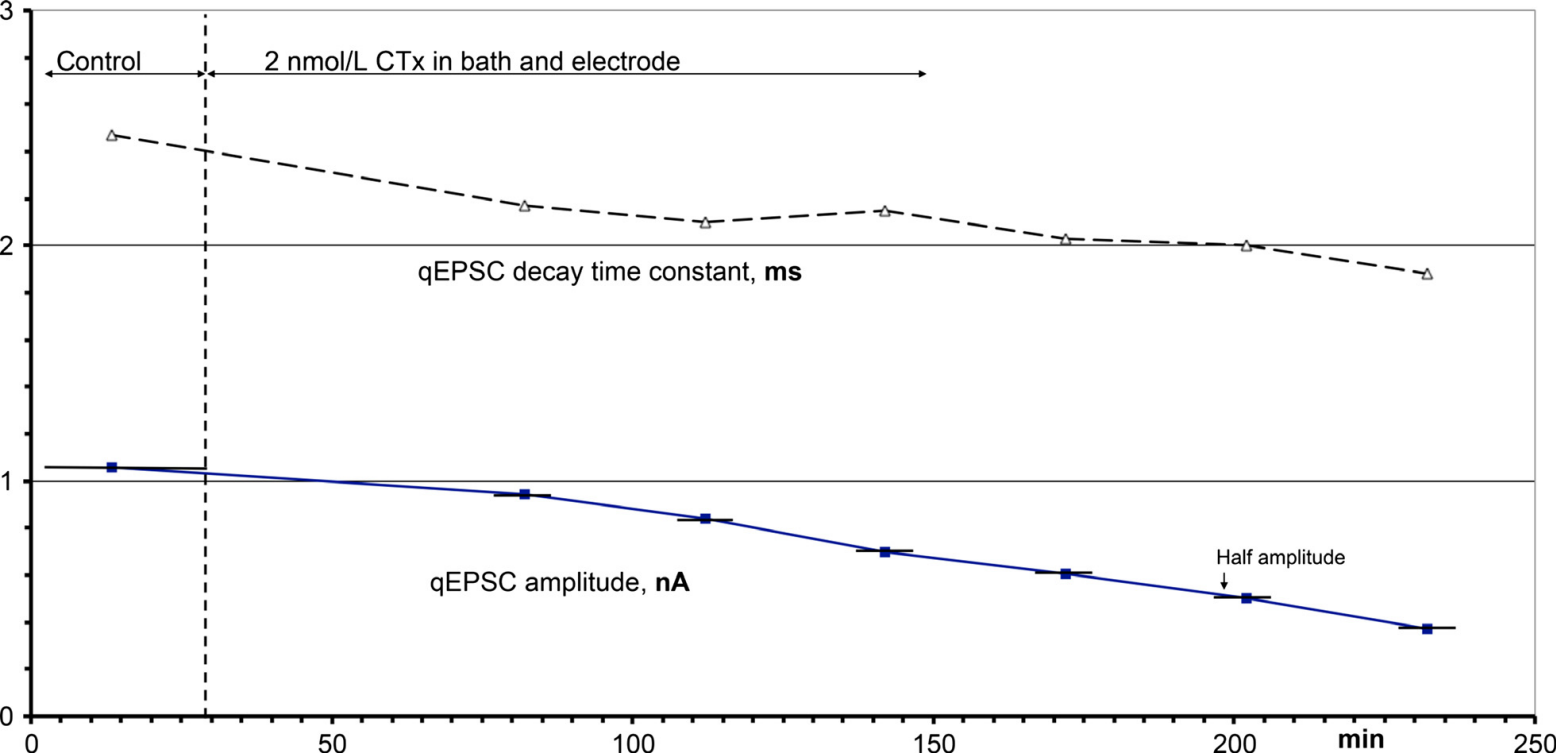
Time course of the effects of 2 nmol L^−1^ CTx on the amplitude and the decay time constant of qEPSCs. After the control period both the perfusate of the electrode and the superfusate of the bath were switched to 2 nmol L^−1^ CTx. The periods in which data were recorded are indicated at the amplitude plot by horizontal bars.

When a very low CTx concentration, 2 nmol L^−1^, was applied for 200 min (Fig. 4), the amplitudes of qEPSCs decreased continuously and with an almost constant slope. The 50% amplitude was reached after 170 min, and the amplitude continued to decrease with no indication of approaching an equilibrium. It could be argued that this result may be an artifact: the tip of the recording electrode might have slowly slipped from the optimal recording position, and the qEPSC amplitude decreased accordingly. Recordings became unstable rarely in our experiments, but then abruptly and not steadily over hours. Anyway, all four further experiments of this type showed the same continuous decline of the qEPSCs, which seems to exclude a progressive failure in recording the qEPSCs (see also Fig. 6B). In Fig. 4 the slope of the decrease in the qEPSC amplitude was 4.0 **×** 10^−5^ s^−1^ relative to the control amplitude, and the average of all five experiments was 4.6 **×** 10^−5^ s^−1^ with a standard deviation of 0.46 **×** 10^−5^ s^−1^.

**Figure 5.**
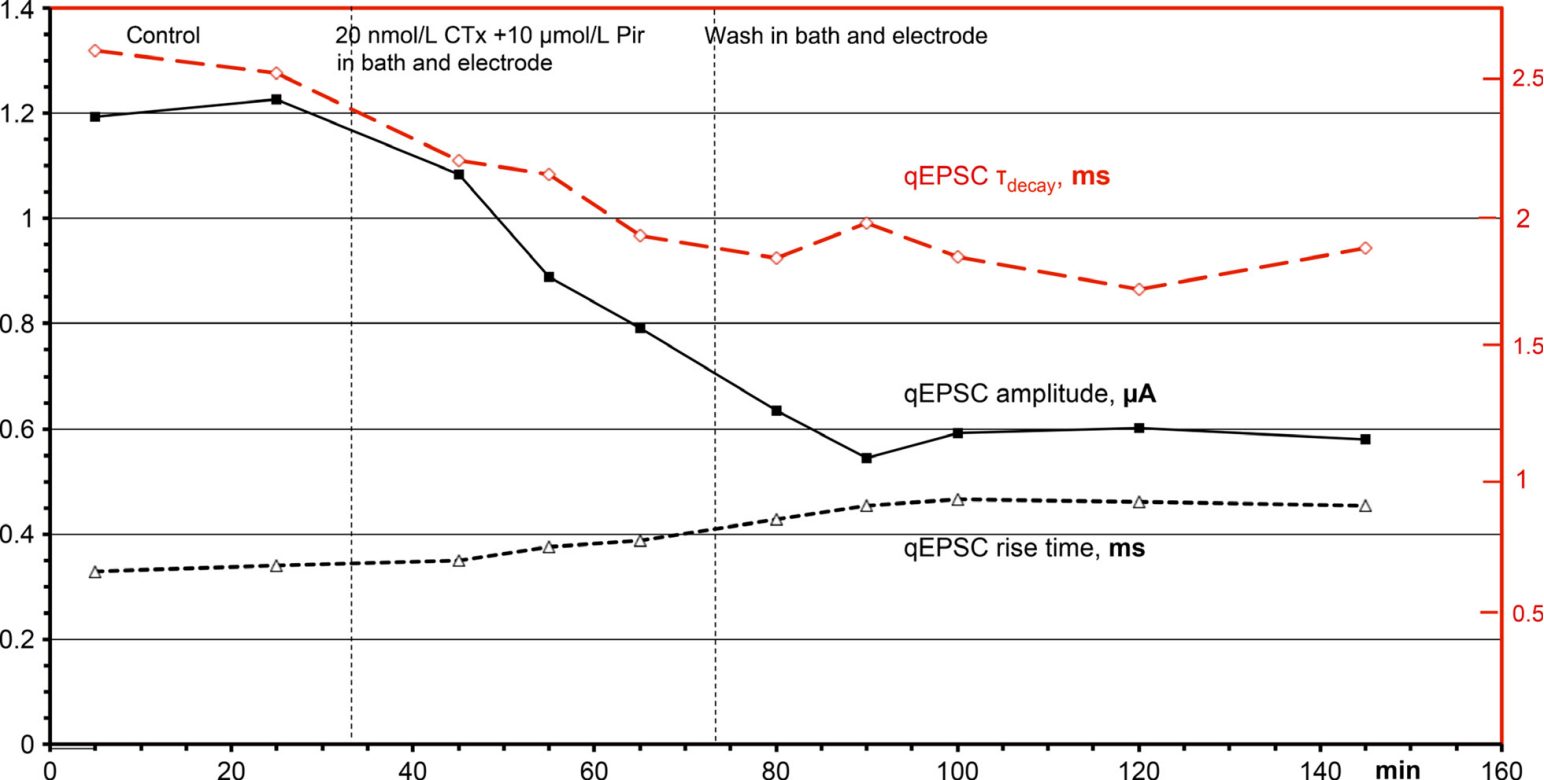
Time course of the effects of 20 nmol L^−1^ CTx + 10 *μ*mol L^−1^ pirenzepine (Pir, blocker of M1 receptors) on amplitude, rise time, and decay time constant of qEPSCs. The drugs were applied for 40 min to the bath and the electrode, similar to the procedure in Fig. 4. Each of the plotted points represented 10 min of recordings (3000 pulses).

In experiments like that of Fig. 3, when the perfusate of the electrode was switched back to control after application of CTx, the amplitudes and rise times of qEPSCs finally recovered, albeit very slowly. This demonstrated the stability of the recordings. In contrast, experiments like that in Fig. 4 suggested possible irreversibility of the effects of CTx. The apparent recovery in Fig. 3 may be due to the experimental conditions: in Fig. 3 only the electrode was perfused with CTx, whereas outside the electrode the bath continued to be superfused with control solution. The partial reversal of the CTx effects after switching the perfusate of the electrode back to control solution may be due to receptors outside the electrode that had no contact with CTx and later diffused into the space below the electrode. To exclude this possibility, in the experiment of Fig. 5 CTx was applied both in the bath and through the electrode, resulting in a reduction in the qEPSC amplitude, an increase in the rise time of the qEPSCs (as in Fig. 3) and further into some shortening of the decay time constant of the qEPSCs, as seen in all such experiments (see Figs. 2, 4 and 5). After 40 min of application, CTx was washed out in both the perfusates of the bath and the electrode. The depression of the amplitude of the qEPSC and the other CTx effects remained constant throughout the washing period. The same results were obtained in 11 similar experiments. A spurious recovery from the effects of CTx seems to be based on nicotinic receptors outside the electrode that had not been exposed to CTx (see Discussion).

**Figure 6.**
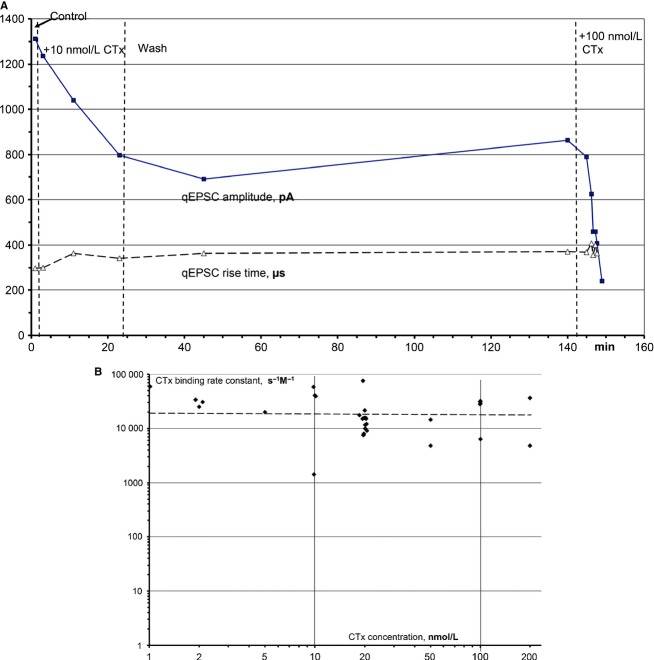
Concentration dependence of the effects of CTx. (A) Initially 10 nmol L^−1^ CTx, and after 1 h of washing 100 nmol L^−1^ CTx were applied through the perfusate of the electrode only. The slopes of the resulting depressions of the qEPSC amplitudes were evaluated. (B) CTx blocking rate constants were plotted against the applied CTx concentrations. The rate constants have been determined from the slopes of depression of qEPSC amplitudes (like in Fig. 6A) that were divided by the CTx concentration. The plot presents values from all 31 experiments of this type. The line is a linear fit of the rate constants. Note the double‐logarithmic scales.

In embryonic nicotinic receptors, low CTx concentrations bind and block only at one, the *αδ*‐site of the nicotinic receptor (Sine et al. 1995). We expect the same to be true for the adult receptor, and then the rate of block by CTx should be proportional to CTx concentration. In Fig. 6A, 10 nmol L^−1^ CTx depressed the qEPSC amplitude at a rate of 0.018 min^−1^. After 2 h of washing and some recovery of the amplitude, 100 nmol L^−1^ CTx depressed the amplitude at a rate of 0.18 min^−1^, astonishingly close to the predicted proportionality. Due to the relatively large variability of the CTx effect, other such experiments showed less or more than proportional depression in relation to changes in the CTx concentration. In order to include results from all 31 such CTx applications, we divided the slopes of amplitude depression by the applied CTx concentration and obtained the binding rate constants of CTx at concentrations from 1 to 200 nmol L^−1^ (Fig. 6B). The values vary widely, but on average the binding rates are almost concentration independent at about 2 **×** 10^4^ s^−1^·mol^−1^ L. They decrease slightly with rising concentrations; if CTx would bind also to the second binding site, the rate of block should increase steeply with the concentration. Thus, at concentrations of at least up to 200 nmol^−1^ L, CTx binds only to one binding site also at the adult nicotinic receptor.

### Presynaptic effects of CTx

As mentioned in the introduction, we found also presynaptic effects of CTx on the release of quanta of ACh. These are unexpected side effects and complications in the study of postsynaptic receptors. We therefore restricted the presentation of presynaptic effects to aspects related to the main theme.

#### Inhibition of release

**Figure 7.**
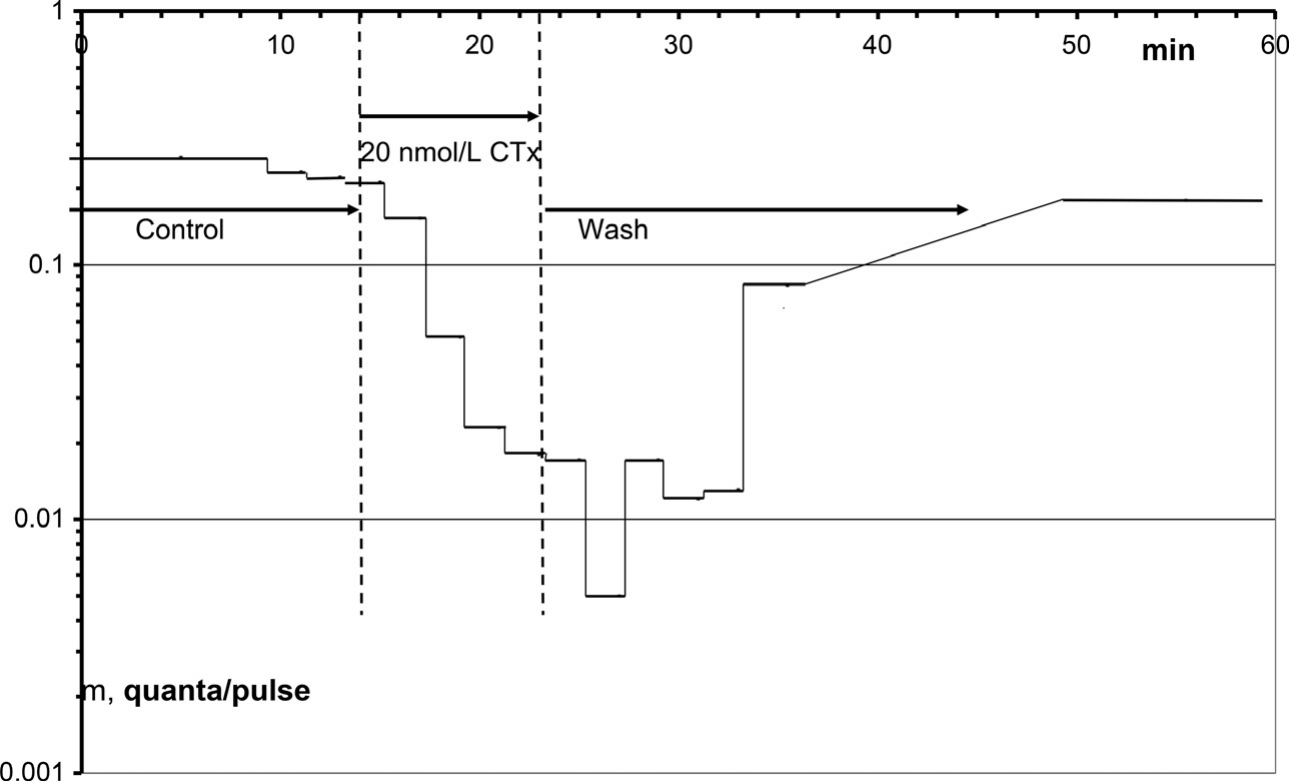
Time course of block and recovery of the rate of quantal release, m, after application of 20 nmol L^−1^ CTx; same experiment as that in Fig. 3. Note the logarithmic scale of the ordinate. The lengths of the horizontal bars represent the duration of the respective measurement.

Figure 3 has shown the reduction in the amplitude of qEPSCs after a short application of 20 nmol L^−1^ CTx and its very slow, if spurious recovery on washing out the CTx. In the same experiment, CTx also inhibited the release of quanta from the nerve terminal (Fig. 7): The number of quanta released per depolarization pulse, m, dropped 50‐fold after 11 min of 20 nmol L^−1^ CTx. The maximal slope of the depression of release was 6.4 **×** 10^5^ s^−1^·mol^−1^ L, similar to the respective slope of the depression of the qEPSC amplitude of 5.4 **×** 10^5^ s^−1^·mol^−1^ L, at the same synaptic site. The maximal depression of release occurred with a delay of 5 min after switching the perfusate of the electrode back to control solution. Different from the postsynaptic inhibition, presynaptic inhibition recovered almost fully with a time constant of about 20 min. In all experiments in which alone CTx has been applied, release was reduced and recovered faster on washing than the simultaneous postsynaptic effects. Often, like in the experiment of Figs 3 and 7, the presynaptic inhibition was more effective than the postsynaptic one.

It might be argued that the described presynaptic inhibition by CTx is really a postsynaptic one, a reduction in some qEPSC amplitudes to a level that could not be resolved. In the experiment of Figs 3 and 7, the maximal presynaptic inhibition occurred at minute 27 with an average qEPSC amplitude of 324 nA. As seen in Fig. 2B, this range of amplitudes is well resolvable – even the rise times and decay time constants were evaluated automatically. The maximal depression of the qEPSC amplitude in Fig. 3 occurred later, at minute 35.5 and 242 nA amplitude. Similarly, in the experiment of Fig. 6 A, 20 min of 10 nmol L^−1^ CTx reduced the amplitude of qEPSCs from a control of 1311 to 642 nA at minute 45. The rate of release (not plotted) dropped from 0.47 to 0.24 quanta/pulse at minute 11, when the qEPSC amplitude still measured 1039 nA. Thus with the employed CTx concentrations and their relatively short periods of application postsynaptic inhibition does not cause the observed inhibitions of quantal release.

**Figure 8.**
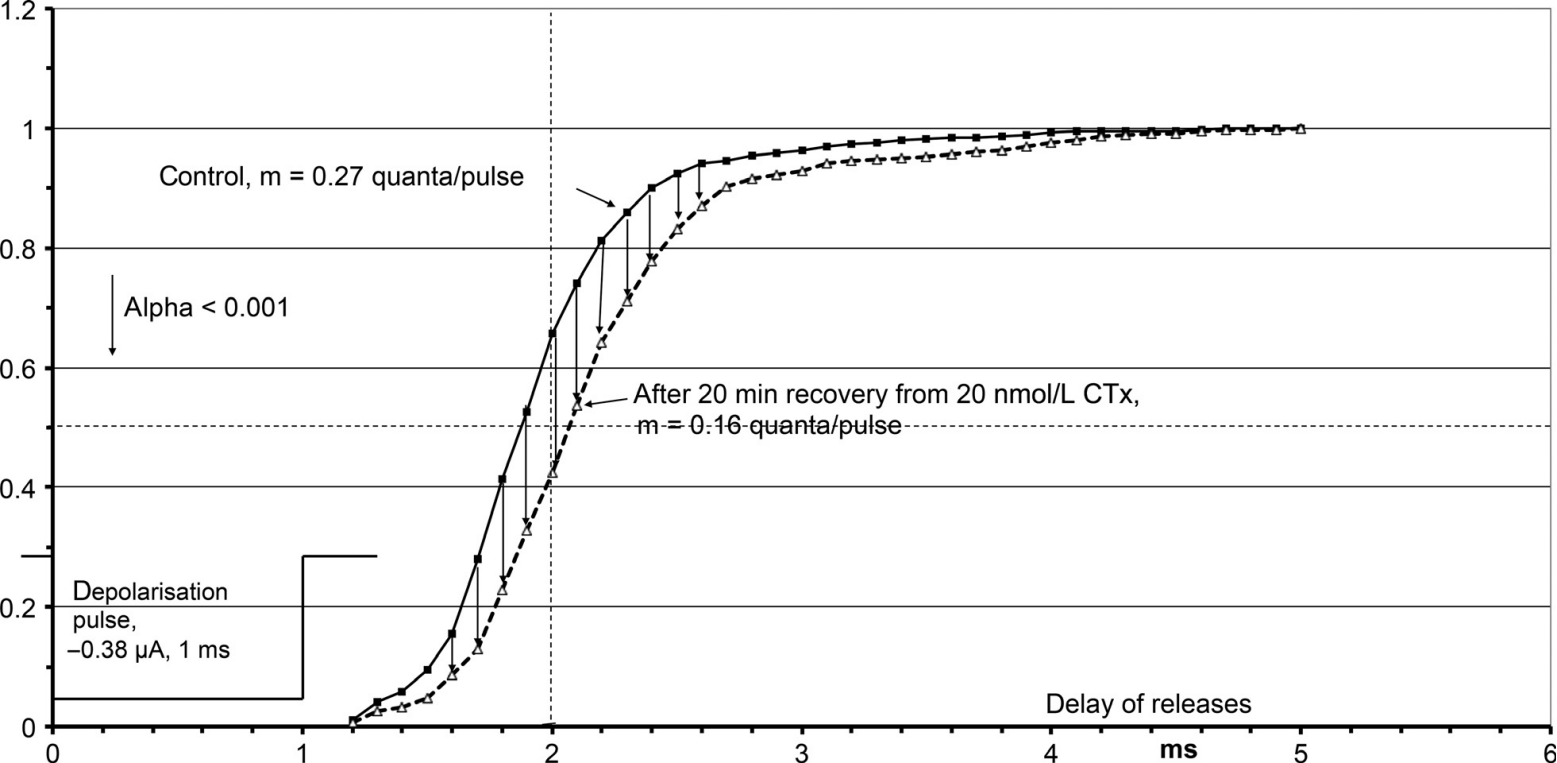
Shifted time course of release after application of 20 nmol L^−1^ CTx. Cumulative plot of delays of quantal releases from the beginning of the pulse, same experiment as that in Figs 3 and 7. The lengthening of the delays is significant with a probability >0.999 (Kolgomorov–Smirnov statistics; Sachs 2003).

**Figure 9.**
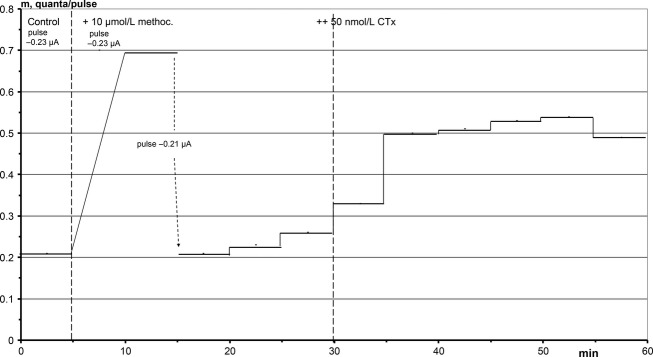
CTx facilitates release after block of muscarinic M2 receptors by 10 *μ*mol L^−1^ methoctramine. The experiment starts with a control, and application of methoctramine through the electrode more than triples the rate of release, m. In order to remain at a low release level, the amplitude of the pulse that triggers release was reduced. After application of 50 nmol L^−1^ CTx release doubled.

**Figure 10.**
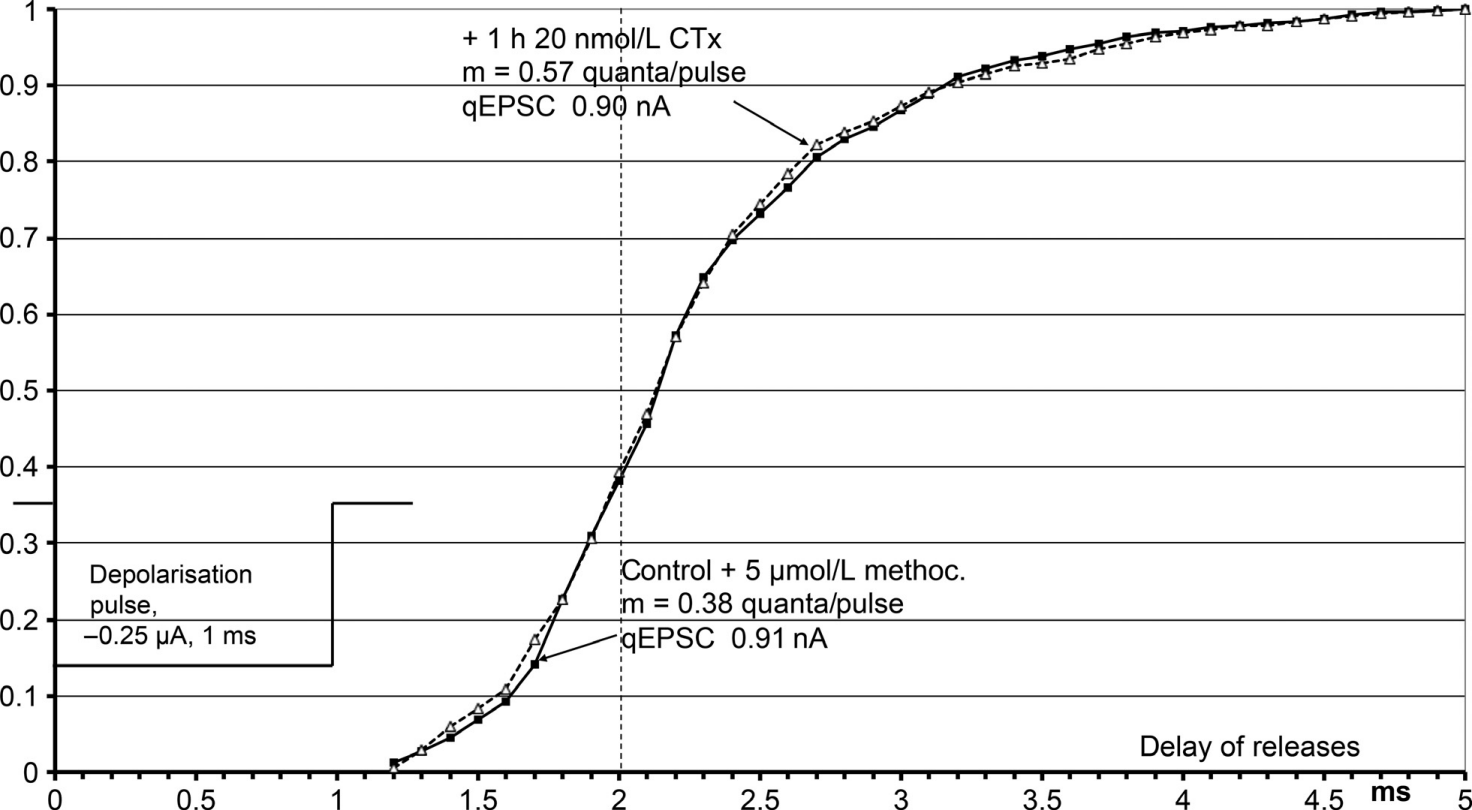
Activation of M1 receptors in presence of methoctramine does not affect the delays of releases. Cumulative plot of delays of quantal releases from the beginning of the pulse (compare Fig. 8).

A well‐known depression of release by the agonist ACh (autoinhibition) is mediated by binding to muscarinic M2 autoreceptors (Slutsky et al. 1999; Minic et al. 2002; Santafé et al. 2003; Nikolsky et al.^2004^). CTx possibly is an agonist at the muscarinic M2 receptors. Dudel (2007) has shown that activation by muscarine of the M2 receptors of mice delayed release. Figure 8 is a cumulative plot of the delays of quantal releases from the start of the 1 ms depolarization pulse in the experiment of Fig. 7. The delays are seen to be increased by about 0.2 ms highly significantly. The effects of CTx in Fig. 8 have been evaluated after 20 min recovery during washing, when most of the presynaptic CTx effects had recovered. The cumulative plot of delays needs large numbers of events that could only be gathered during recovery. Final proof for CTx to be an agonist at the muscarinic M2 receptors that mediate presynaptic autoinhibition is given in the next paragraph: if methoctramine, the specific blocker of M2 receptors, is applied with CTx, there is no inhibition of release (Figs 9 and 10).

Also in ‘embryonic’ mouse diaphragms CTx inhibited release, but this presynaptic inhibition has not been studied in detail, and no blockers of muscarinic receptors have been employed.

#### Facilitation of release

As mentioned in the introduction, release of ACh quanta from synaptic nerve terminals on vertebrate muscles is regulated not only by inhibitory M2 but also by facilitatory M1 receptors. When muscarine is applied to an endplate (Dudel 2007), the inhibition via M2 is much stronger than the facilitation via M1, and the latter is masked. Facilitation can be demonstrated only if the M2 receptors are blocked by methoctramine, a specific blocker of M2 receptors.

The experiment of Fig. 9 started with a control release of 0.21 quanta/pulse. When 10 *μ*mol L^−1^ methoctramine was added to the perfusate of the electrode, release tripled. Such increases in release occur usually on application of methoctramine to an endplate. The block of M2 receptors apparently unmasks a latent inhibition of release by a low ambient ACh concentration that activated M2 receptors (It cannot be excluded that methoctramine directly activates M1 receptors). The initial facilitation in Fig. 9 was uncommonly strong, and in order to remain at a low level of release, the amplitude of the depolarizing pulse was reduced, resulting in a rate of release of again 0.21 quanta/pulse. Release continued to rise slowly.

At 30 min, 50 nmol L^−1^ CTx was added to the perfusate, and release about doubled again within 10 min, presumably due to the activation of M1 receptors by CTx. This conclusion is supported by the experiment in Fig. 10. While presynaptic inhibition via M2 receptors delayed release (Fig. 8), facilitation via M1 receptors did not, as shown also for muscarine (Dudel 2007).

To our surprise, 10 *μ*mol L^−1^ methoctramine reduced the amplitudes of the postsynaptic currents from 1.6 nA to 1.2 nA, and simultaneously shortened the time constants of decay of the qEPSCs from 1.74 ms to 1.0 ms (not plotted in Fig. 9), and similar reductions were seen in four other such experiments. These findings might indicate that relatively high concentrations of methoctramine block the nicotinic receptor like CTx does. Further experimental investigations will be necessary to substantiate this suggestion.

## Discussion

### Postsynaptic effects of CTx

In patch‐clamp studies of the effects of CTx on embryonic muscle (Stock et al. 2014), complete block of the *αδ* binding site abolished the long bursts of channel openings that generate the normal EPSCs. Binding of ACh at the *αγ*‐site elicited single openings and short bursts of a few openings. In comparison to the long bursts of the controls they would generate almost negligible and very short synaptic currents. On adult muscle, we have used much lower CTx concentrations than Stock et al., but still had to stop the application of CTx before the EPSCs disappeared (usually due to block of quantal release). The block of the *αδ* binding sites of the nicotinic receptors certainly never was complete. The decay time constant of qEPSCs corresponds to the burst duration (Magleby and Stevens 1972). CTx decreases the decay time constants obviously due to a contribution of currents from short bursts generated by the binding of ACh to the *αε*‐site. The distribution of decay time constants in Fig. 2 D shows a short component in addition to the one produced by the not yet blocked long bursts. The average duration of the short component should be larger than that of the ‘short bursts’, as most receptors still did not bind CTx and produced ‘long bursts’.

The *αδ* binding site is preferred over the other for the binding of ACh and CTx (Fig. 1). Consequently, binding of ACh to the *αε*‐site should be slower than that to the *αγ*‐site, and short bursts arising from CTx‐blocked receptors should be slightly delayed relative to the long bursts generated without the block. Such delayed bursts could be responsible for the lengthened rise times of the partially CTx‐blocked qEPSCs.

In adult muscles, nanomolar concentrations of CTx effectively reduced the amplitudes of qEPSCs: continuous application of 2 nmol L^−1^ CTx led to almost complete block with a constant slope (Fig. 4). The average slope was 4.6 **×** 10^−5^ s^−1^, and this corresponds to a blocking rate of CTx of 2.2 **×** 10^4^ s^−1^·mol^−1^ L. With wide variations, the blocking rates were almost constant from 1 to 200 nmol L^−1^ CTx, with an average of 2.3 **×** 10^4^ s^−1^·mol^−1^ L (Fig. 6B). These concentration‐independent rates indicate block by binding at one site, presumably the *αδ*‐site. The blocking rate of the nicotinic receptor by CTx is 20 times lower than that of the competitive block by (+) tubocurarine (Bufler et al. 1996a) and 100 times lower than that of the open channel block by procaine (Bufler et al. 1996b).

In experiments like that of Figs 2 and 3, when CTx was applied for a certain period of time through the electrode alone, after washing out the CTx the amplitude of the qEPSCs slowly recovered. If, however, CTx was applied simultaneously through the electrode and in the superfusate of the muscle, that is, inside and outside the electrode, washing out the CTx did not elicit a recovery of the qEPSC amplitude (Fig. 5). Also the rise times of the qEPSCs and their decay time constants remained at the level attained by the application of CTx (Fig. 5). Obviously, the superfusion of the muscle with CTx prevented that, on washing, a surplus of unliganded receptors diffused within the membrane of the muscle cell into the space below the electrode and feigned unbinding of CTx from some receptors. Diffusion of receptors within the membrane is very complex; some receptors move freely, others are fixed to the cytoskeleton (Tardin et al. 2003; Gerrow and Triller 2010; Czöndör et al. 2012).

If ‘recovery’ by receptor diffusion is discounted, is there recovery at all? Is binding of CTx to the adult receptor irreversible? In experiments like that in Fig. 4, continuous application of 2 nmol L^−1^ CTx for hours depressed the qEPSCs with a constant average slope of 4.6 **×** 10^−5^ s^−1^ with no indication of approaching an equilibrium even after passing the reduction to half amplitude. If there should be an equilibrium, it could not be measured with the present experimental setup. It would lie certainly much below 1 nmol L^−1^ CTx. For the embryonic mouse muscle, Sine et al. (1995) reported an IC_50_ of CTx of 3 nmol L^−1^. Biochemists reported IC_50_ values for the binding of CTx to embryonic/juvenile nicotinic receptors of 1.5 nmol L^−1^ (Groebe et al. 1995) to about 20 nmol L^−1^ (Luo and McIntosh 2004). Under our experimental conditions, apparently the substitution of the *ε*‐ for the *γ*‐subunit in the adult receptors substantially increased the affinity for CTx of the *αδ*‐site of the adult receptor; possibly the binding of CTx to adult nicotinic receptors is even irreversible.

### Presynaptic effects of CTx

To our surprise, CTx activated the mechanisms of auto‐inhibition and ‐facilitation of release in the motor nerve‐muscle synapses. Autoinhibition occurs in many types of synapses (Starke et al. 1989; Altman et al. 1999) and is well known to operate in the brain and in the peripheral sympathetic nervous system. It seems to serve as a brake when the ambient transmitter concentration around the nerve terminal rises above a certain low level. In cholinergic motor synapses, muscarinic presynaptic ACh receptors modulate quantal ACh release (North et al. 1985; Slutsky et al. 1999; Santafé et al. 2003, 2006; Nikolsky et al. 2004). When, for example, 20 *μ*mol L^−1^ muscarine is applied to the synapse, M1 receptors facilitate release and M2 receptors potently inhibit release (Dudel 2007). Parnas et al. (2005) suggest that at the resting potential activation of M2 receptors by a sufficiently high ambient ACh concentration fully blocks release, and that this block is relieved by depolarization of the terminal.

CTx primarily inhibits release, at concentrations as low as 10 nmol L^−1^, and does so more potently at higher concentrations (Fig. 6 A). Methoctramine is a specific blocker of muscarinic M2 receptors, and it prevents inhibition of release by CTx (Fig. 9). It even turns inhibition into facilitation. These effects of methoctramine indentify the presynaptic CTx receptors as muscarinic M2 and M1 receptors that are about 1000 time more sensitive to CTx than to muscarine. Muscarine not only inhibits release, but also slightly delays the time course of release (Dudel 2007). The same shift of the time course of release was also elicited by CTx (Fig. 8), and the shift was absent when the M2 receptors were blocked by methoctramine (Fig. 10). This supports the identification of the presynaptic CTx receptors as M1 and M2 receptors.

The polyamine methoctramine was primarily employed in order to identify the presynaptic receptors as muscarinic autoreceptors. However, high concentrations of methoctramine reduced the amplitude and the decay time constant of qEPSCs. These actions of methoctramine appeared to be similar to the block of nicotinic receptors by CTx. As many polyamines, methoctramine is thought to be an open channel blocker at high concentrations (Re et al. 1993; Bixel et al. 2000; Rosini et al. 2002; Nikolsky et al. 2004), cutting off the length of bursts of channel openings by entering the channel at a high rate.

Could CTx also shorten the decay of qEPSCs by open channel block? In the classical open channel block of nicotinic receptors, high ACh concentrations blocked the open channel with very short intermitted closings that prolonged the duration of bursts (Neher and Steinbach 1978; see Parzefall et al. 1998). The local anesthetic procaine produces open channel block with a rate of 5 **×** 10^6^ s^−1^·mol^−1^ L and unbinds with a rate of 350 s^−1^, that is, on average after 3 ms (Bufler et al. 1996a; Dudel et al. 1999)). One millimolar procaine then would start a block with a rate of 2 **×** 10^3^ s^−1^, and would cut off a burst on average after 0.5 ms open time. This would result in a short large component of the qEPSC followed by a slowly decreasing tail of current due to reopenings of the channel. (+)‐tubocurarine has a similar rate of binding to the open channel and shortens qEPSCs accordingly, but the rate of unbinding is only 0.8 s^−1^ (Bufler et al. 1996b). The open channel block will last more than a second, and there was no tail of the shortened EPSC due to reopenings of the channel. It appears that the open channel block by methoctreamine is similar to that by tubocurarine.

For a possible open channel block by CTx, one has to consider the rate of binding to the open channel and the CTx concentration. The rate of binding remains unknown, but it should be lower than the rates for procaine or tubocurarine. CTx is a much larger and more rigid molecule than the latter, and one may doubt whether CTx would fit into the channel at all. Let us assume a rate of binding of CTx to the open channel of 10^6^ s^−1^·mol L^−1^. At 2 **×** 10^−7^ mol L^−1^, the highest concentration of CTx used here, the binding rate to the open channel would be 0.2 s^−1^, that is, block after 5 sec – there is no open channel block by CTx.

Even with the highest CTx concentration used in Stock et al. (2014), 2 **×** 10^−4 ^mol L^−1^, binding to the open channel would occur after 5 ms.

CTx is a very potent poison at pre and postsynaptic receptors in the nervous system. Possibly it does not only affect the motor synapses of vertebrates, but also other synaptic systems with nicotinic and/or muscarinic receptors.

The group of substances that appear in this study, ACh, CTx, methoctramine, muscarine, nicotine, procaine, and tubocurarine, all bind to some ACh receptor. It is not surprising that their specificity for binding to a certain receptor type is limited. For instance, in Fig. 9 methoctramine applied to the control elicited a more than threefold increase in release. Maybe the control was inhibited due to activation of M2 receptors by a low ambient ACh concentration, and this inhibition was relieved by methoctramine. But it cannot be excluded that methoctramine activated faclitatory M1 receptors.

## Acknowledgements

We thank Drs. Manfred Heckmann and Stephan Hallermann for comments on the manuscript, and Mrs. Hongbo Jia, Mrs. Christine Karrer and Mrs. Frauke Köhler for technical assistance.

## Conflict of Interest

None declared.
